# The Role of the Intestine in the Development of Hyperuricemia

**DOI:** 10.3389/fimmu.2022.845684

**Published:** 2022-02-24

**Authors:** Hui Yin, Na Liu, Jie Chen

**Affiliations:** ^1^ Department of Rheumatology and Clinical Immunology, Jiangxi Provincial People’s Hospital, The First Hospital of Nanchang Medical College, Nanchang, China; ^2^ Department of Rheumatology and Clinical Immunology, Jiangxi Provincial People’s Hospital Affiliated to Nanchang University, Nanchang, China

**Keywords:** gout, hyperuricemia, ABCG2, SLC2A9, intestinal flora

## Abstract

Gout is a common inflammatory arthritis caused by the deposition of sodium urate crystals in the joints. Hyperuricemia is the fundamental factor of gout. The onset of hyperuricemia is related to purine metabolism disorders or uric acid excretion disorders. Current studies have shown that the intestine is an important potential organ for the excretion of uric acid outside the kidneys. The excretion of uric acid of gut is mainly achieved through the action of uric acid transporters and the catabolism of intestinal flora, which plays an important role in the body’s uric acid balance. Here we reviewed the effects of intestinal uric acid transporters and intestinal flora on uric acid excretion, and provide new ideas for the treatment of hyperuricemia and gout.

## Introduction

Gouty arthritis is an inflammatory disease caused by the deposition of sodium urate crystals in and around the joints caused by long-term hyperuricemia (1). Uric acid is the final product of human purine metabolism. Uric acid in the human body maintains a dynamic balance under the action of the liver, kidneys and intestines. When the balance is lost, the serum uric acid level will increase (2). Hyperuricemia (>6.8mg/dL) is the main cause of gout and a risk factor for cardiovascular disease, kidney disease, metabolic syndrome and other diseases (3–6). Under physiological conditions, two thirds of uric acid is excreted from the kidneys and one third is excreted through the intestines (7). Hyperuricemia was divided into overproduction of uric acid and insufficient excretion (8, 9). In recent years, the hypothesis of “kidney overload” is increasingly recognized, suggesting that the types of hyperuricemia should be changed to renal excretion disorders and renal overload types, which including insufficient extrarenal excretion and excessive uric acid production (10). The extrarenal excretion of uric acid is mainly achieved through the intestinal tract. The current uric acid lowering drugs mainly include three categories: inhibiting the production of uric acid, promoting the dissolution of uric acid, and promoting the excretion of uric acid in the kidneys (11). Several drugs can inhibit the production of uric acid, such as allopurinol, febuxostat, and topiroxostat. Allopurinol reduces the synthesis of uric acid by inhibiting xanthine oxidase. However, allopurinol hypersensitivity syndrome (AHS), in which allopurinol has a fatal risk, warrants attention, and the incidence of AHS is higher in Asians, especially Han people (12). As a non-purine xanthine oxidase inhibitor, febuxostat is better than allopurinol in inhibiting the production of uric acid. However, it has been reported that febuxostat can increase the mortality of cardiovascular events in patients with gout. Other adverse reactions include muscle pain, elevated liver enzymes, etc. (11, 13). Recombinant uricase that promote the dissolution of uric acid, such as rasburicase, pegloticase, and pegloticase have a higher probability of infusion-related reactions such as rash, headache, and dyspnea (14, 15). Benzbromarone, probenecid, and lesinurad can promote the excretion of uric acid in the kidney. Benzbromarone inhibits the reabsorption of uric acid in the renal tubules to achieve the purpose of lowering uric acid, which can lead to the formation of uric acid kidney stones and liver toxicity (16). Probenecid and lesinurad reduces uric acid reabsorption by inhibiting the activity of renal uric acid transporter, but adverse reactions may occur in various systems (17). A clinical trial has shown that lesinurad monotherapy treats increased serum creatinine and the occurrence of renal-related adverse events (18). At present, the target of drugs for promoting uric acid excretion is mainly concentrated in the kidneys, which will increase the burden on the kidneys, especially for patients with chronic kidney disease. As the largest organ of the human body, the intestine has a huge potential for uric acid excretion, and it is hoped that it will become a safer and more effective target organ for lowering uric acid drugs. This article reviews the metabolic pathways of uric acid in the intestines and the possible therapeutic targets derived therefrom.

## Metabolic Pathway of Uric Acid

**Figure 1:**
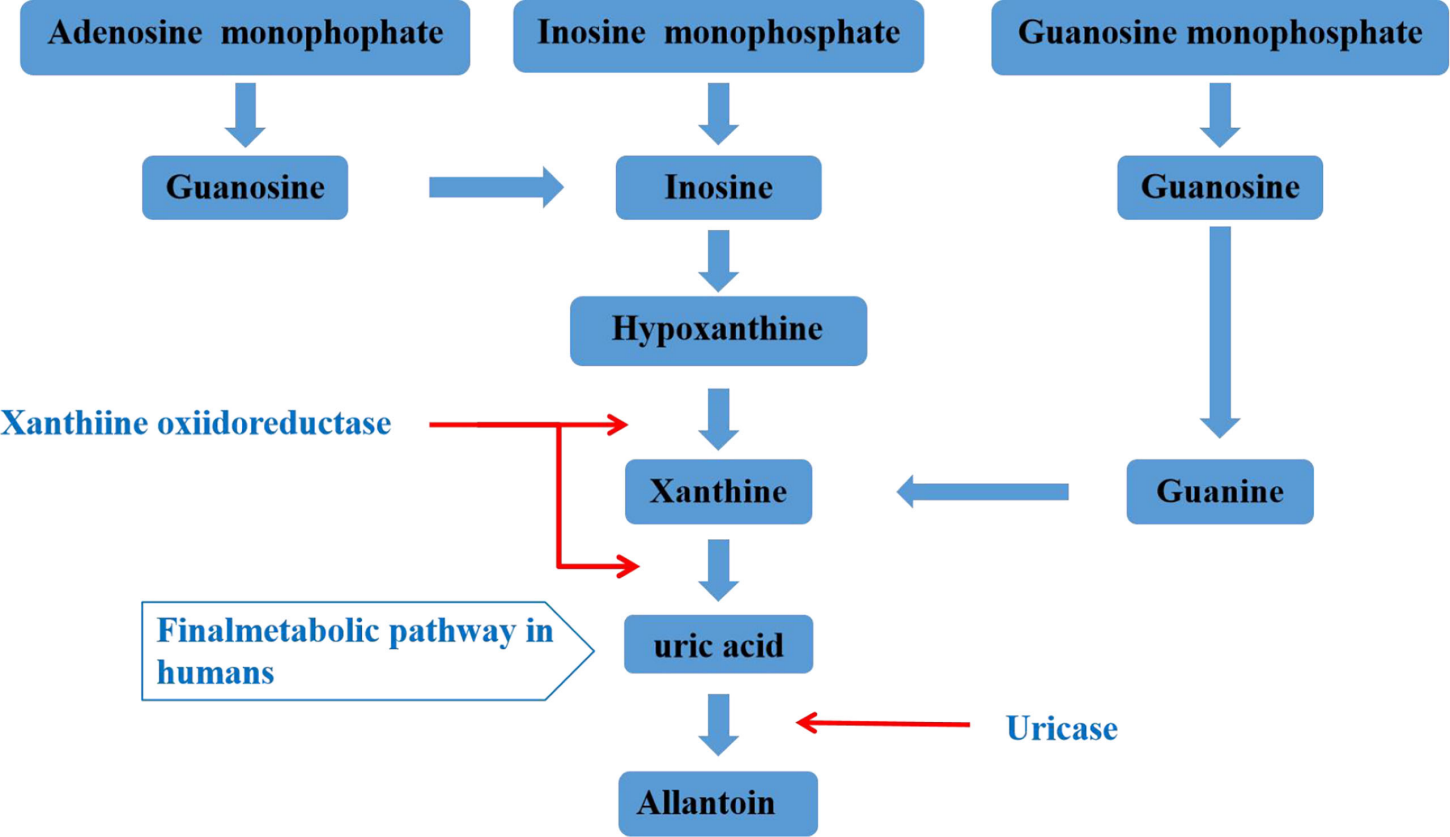
Metabolism of uric acid.

**Figure 2:**
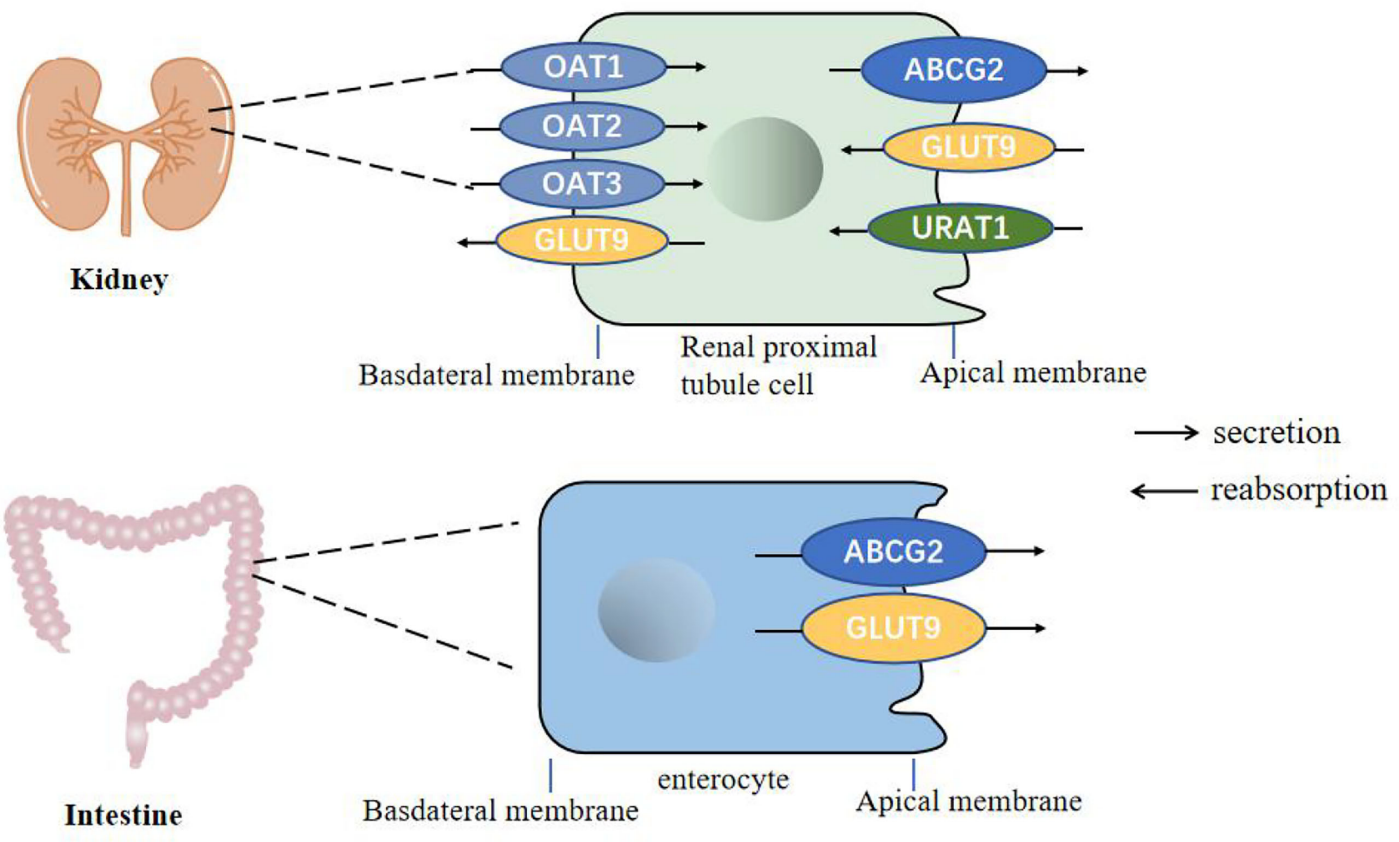
Urate transporters in the kidneys and intestine in humans. GLUT9 and URAT1 on the proximal tubule cells mediate renal urate reabsorption, while GLUT9 on the enterocytes mediate urate excretion in the intestine. ABCG2 is involved in urate export in both the intestine and the kidney. OAT1, OAT2 and OAT3 are present on the basolateral membrane of renal proximal tubule cells and mediates urate secretion.

Uric acid is mainly synthesized in the liver, and a small amount is produced in the small intestine. It is the final product of human purine metabolism. Purine nucleotides generate adenosine, inosine and guanosine under the action of adenosine deaminase, adenosine is deaminated to form inosine, and inosine and guanosine are further converted into hypoxanthine and guanine, hypoxanthine Purine forms xanthine under the action of xanthine oxidase, and guanine deaminates to form xanthine. Xanthine is oxidized again by xanthine oxidase and finally produces uric acid (**Figure 1**) (19, 20). In most mammals, uricase can oxidatively degrade uric acid into the soluble compound allantoin. In the process of human evolution, the gene encoding uricase has undergone inactivation mutations, resulting in a lack of uricase (21). It has been shown that two-thirds of the uric acid in the human body is excreted from the kidneys, one-third is excreted from the intestines and bile, and the proportion of uric acid excreted by bile is very small. The kidney regulates the excretion of uric acid through the reabsorption and secretion of proximal tubules, and this process is mainly achieved through uric acid transporters (22, 23). Uric acid transporter dysfunction plays an important role in the pathogenesis of hyperuricemia. Genome-wide association studies (GWAS) found that many genes related to hyperuricemia and gout. The genes encoding proteins referred to as uric acid transporter (24, 25), including URAT1, OAT4 and SLC2A9 that mediate the reabsorption of uric acid, and transporters such as ABCG2, OAT1, OAT2, OAT3, and MRP4 that mediate the secretion of uric acid (**Figure 2**) (26).

## The Effect of Intestinal Uric Acid Transporters on Level of Uric Acid

The blood uric acid concentration is related to a variety of transporter-encoding genes. Among them, the uric acid transporter in intestinal epithelial cells transports uric acid from the blood to the intestinal lumen, which involves the participation of multiple transporters, mainly ABCG2 and SLC2A9 (27).

### ABCG2

ABCG2, also known as breast cancer resistance protein (BCRP), containing 1 transmembrane domain and 1 ATP binding domain. ABCG2 gene is located at the gout susceptibility site of chromosome 4q (28), and expressed on the apical membrane of cells in various tissues such as the intestine, liver and kidneys (29, 30). ABCG2 is a high-volume uric acid transporter. More and more studies have found that ABCG2 plays an important role in intestinal uric acid excretion and the pathogenesis of hyperuricemia. The dysfunction of ABCG2 reduces the excretion of uric acid in the intestinal tract, resulting in increased blood uric acid levels.

Dehghan et al. first proposed the correlation between the ABCG2 gene and uric acid level and gout (31). It has been found that the uric acid content was significantly reduced by 75.5% in ABCG2 expressed oocytes when compared with the control (32). Consistently, the serum uric acid level is increased in ABCG2 gene knockout mice in a established a hyperuricemia mouse model by using the urinase inhibitor potassium oxonate. Interestingly, the intestinal uric acid clearance rate is significantly reduced, while the renal uric acid excretion compensatoryly increases (10). To explore the distribution of uric acid in the body, a mouse model of hyperuricemia with 14 C-labeled uric acid was established. The results showed that the expression of uric acid in the intestine was second only to that in the kidney, and ABCG2 transporter inhibitor elacridar can significantly reduce the clearance rate of uric acid in the intestine (33). The above data show that inhibiting ABCG2 expression can reduce the excretion of uric acid in the intestine, confirming that intestinal ABCG2 dysfunction is one of the pathogenesis of hyperuricemia.

The expression of ABCG2 is regulated by a variety of transcription factors and hormones, and the mechanism is still unclear. Previous study has shown that toll-like recepter 4 (TLR4)-NLRP3(NOD-,LRRand pyrin domain-containing 3) inflammasome and phosphatidylinositol 3-kinase protein kinase B (PI3K/Akt) signaling pathway up-regulates the expression of ABCG2 through PDZK1 on the HT-29 and Caco-2 cells membrane (34, 35). Consistently, ABCG2 gene mutation has the greatest impact on human serum uric acid levels through affecting its protein expression level and uric acid transport efficiency. Q141K and Q126K are the two variants of ABCG2 that cause hyperuricemia (36). The Q141K variant causes a 54% reduction in ABCG2 uric acid excretion, and the latter almost loses its transport function (37). Targeted drugs for ABCG2 mutations have been reported, Woodward et al. found histone deacetylase (HDAC) inhibitor, namely 4-phenylbutyric acid, can correct the conformation of 141K ABCG2 mutant protein and restore its function (38). Uric acid-lowering drugs for ABCG2 dysfunction are expected to become a new treatment direction.

### SLC2A9

The SLC2A9 gene is located on human chromosome 4p and encodes glucose transporter 9 (GULT9), which is expressed in the liver, kidney, small intestine and chondrocytes. A large number of studies have found that SLC2A9 has a strong correlation with uric acid levels (39). SLC2A9 is a voltage-dependent high-volume uric acid transporter, which is involved in the reabsorption of uric acid (40, 41). The genetic variation of the SLC2A9 locus is quite complex. Many other SNPs that are closely related to gout, such as rs4447863, rs737267, rs13129697, rs6449213, rs1014290, rs6449213, rs737267, and rs16890979 (31, 39). A meta-analysis showed that rs3733591 may be a protective SNP in the Caucasian population, while it is a cause for the Asian population (42). Therefore, SLC2A9 mutations may mediate the onset of gout and can become a target for the treatment of gout.

It has shown that SLC2A9 is abundantly expressed in intestinal epithelial cells, especially jejunum and ileum, and is mainly located on the top and basolateral membrane of intestinal epithelial cells, which suggests that SLC2A9 may also mediate the excretion of uric acid from the intestine. Actually, the intestinal cell-specific SLC2A9 gene knock-out mice had elevated serum urate levels, and the mice lacking the SLC2A9 gene were prone to metabolic syndrome (high uric acid, hypertension, hyperglycemia, hyperlipidemia), indicating that SLC2A9 mediates the excretion of uric acid from the intestine (43). However, the mechanism of uric acid metabolism mediated by SLC2A9 in the intestinal tract needs to be further studied.

Studies have shown that the expression of nuclear factor receptor HNF4α can up-regulate the expression of SLC2A9 (44). Peroxisome proliferator-activated receptor PPARγ is a ligand-regulated transcription factor that participates in various pathophysiological processes, including metabolism, inflammation, and tumorigenesis (45). It was also found that the activation of PPARγ can induce the expression of SLC2A9 in the ileum and jejunum. However, the specific regulation mechanism of SLC2A9 in intestinal uric acid excretion needs further study.

### Other Intestinal Uric Acid Transporters

In addition, other intestinal uric acid transporters are also involved in the regulation of serum uric acid levels. A Meta-analysis pointed out that SLC16A9 is related to human serum uric acid concentration (46). It was found that the common rs2242206 mutation of SLC16A9 increased the risk of renal non-low excretion overload hyperuricemia, suggesting that SLC16A9 plays a role in intestinal uric acid excretion (47). Besides, SLC17A4 protein exists in the apical membrane of small intestinal epithelial cells and transports various organic anions including urate, confirming that SLC17A4 is related to serum uric acid levels (48).

## The Influence of Intestinal Flora on Uric Acid and Gout

**Figure 3:**
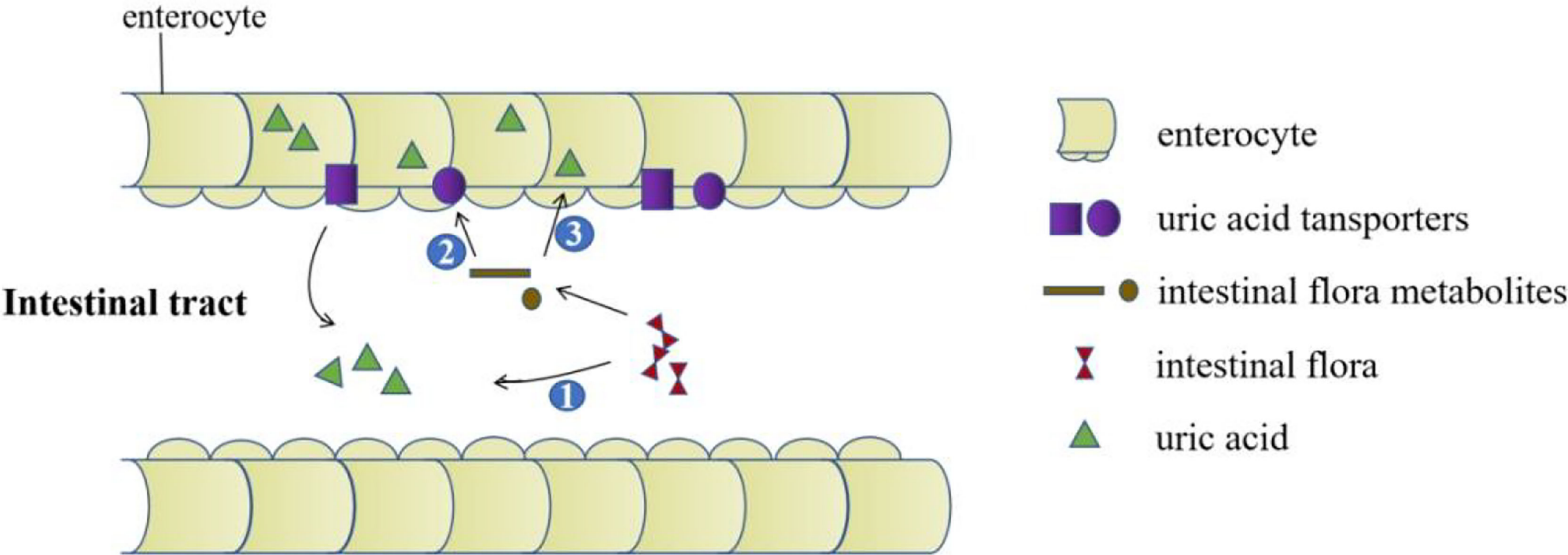
The role of the intestinal tract in uric acid excretion. The intestinal flora decomposes the uric acid directly. Intestinal flora metabolites upregulate the expression of uric acid transporters. Metabolites produced by intestinal flora promote the excretion of uric acid by providing energy for intestinal epithelial cells.

Intestinal microbes are composed of various microorganisms such as bacteria, fungi and viruses in the intestine. At least 100 trillion bacteria in the intestinal microecosystem live in the human intestines. They participate in host metabolism, immune regulation, and maintenance of internal environment homeostasis, etc. (49–51). Increasing evidences show that there are differences in the distribution of intestinal flora between gout patients and healthy people (52, 53). Studies have found that supplementing probiotics can improve uric acid levels. It is promising that probiotics may become a new direction for the treatment of gout and hyperuricemia (54–57). Current research suggests that the impact of intestinal flora on gout is mainly achieved through the following three aspects: participation in purine metabolism and decomposition of uric acid to reduce uric acid levels; metabolites produced by intestinal flora promote the excretion of uric acid (**Figure 3**); participation in immune-inflammatory regulation of gout.

### Intestinal Flora Is Involved in the Catabolism of Uric Acid

It has been shown that intestinal bacteria can decompose uric acid (58). In addition to the intestinal flora participating in the biosynthesis of various substances including essential amino acids, some symbiotic bacteria in the intestine, such as lactobacillus and pseudomonas, which can express uricase, allantoicase, and allantoinase participating in the breakdown of uric acid (59, 60). Under the action of the flora, the uric acid in the intestines eventually produces oxalate and glycine, which provide carbon and nitrogen to the body (61, 62). Lactic acid bacteria isolated from sauerkraut exert the ability to degrade inosine and guanosine, the two key intermediates of purine metabolism. In addition, gavage of specific strains could effectively reduce the serum uric acid level in hyperuricemia rats (63).

### Metabolites of the Intestinal Flora Promote the Excretion of Uric Acid

The intestinal flora can produce some small molecular metabolites that affect host metabolisms, such as short-chain fatty acids (SCFAs), taurine, succinic acid, lipopolysaccharide, acetic acid, butyric acid, and propionic acid (64, 65). It has shown that the types and numbers of the intestinal flora of gout patients and healthy people are significantly different through the 16S rRNA sequencing (66). Further study showed that butyrate-producing bacteria in gout patients decreased by metagenomic analysis, indicating that butyrate may promote intestinal uric acid excretion (67). Besides, a study also showed that the content of glucose, acetic acid, butyric acid in the stool of patients with gout is different from that of the control group (53), and these metabolites are involved in energy metabolism (68, 69), which can provide energy for intestinal epithelial cells and participate in the excretion of uric acid.

### Regulation of Gut Microbiota Metabolites in the Gout Inflammation

As a metabolite of intestinal flora, SCFAs can regulate the function of intestinal epithelial cells, alleviate inflammation and maintain intestinal mucosa homeostasis (70). Previous study showed that the intake of dietary fiber will increase SCFAs and regulate related immune responses (71). Interestingly, high-fiber dietary feeding alleviates the inflammatory response induced by monosodium urate (MSU) through SCFAs production (72). Consistently, a clinical research observation also found that increasing intake of dietary fiber can reduce the symptoms of gout (73). It is currently believed that SCFAs mainly exert their biological effects through the activation of G-protein-coupled receptors (GPCRs) GPR41, GPR43, and GPR109α in intestinal epithelial cells (74, 75). Another intestinal metabolite butyrate also exerts anti-inflammatory effect through GPR43 and GPR109α on macrophages (76, 77). In addition, as an HDAC inhibitor, butyrate can inhibit the acetylation of a variety of proteins in the NF-κB signaling pathway family, thereby reducing IL-1β, IL-2, IL-6, TNFα, and other pro-inflammatory factor releases (78, 79). Furthermore, Angélica et al. found that acetic acid induces caspase-dependent neutrophil apoptosis by inhibiting the NF-κB pathway and promotes the production of IL-10, TGF-β, and annexin A1 to alleviate the inflammatory response (72). These studies suggest that intestinal products also play an important role in the regulation of gout inflammation resulted from the macrophage and neutrophil activation.

## Crosstalk Between Intestine Flora and Uric Acid Transporters

It has shown that intestinal flora has a regulatory effect on expression of uric acid transporters. Anserine, a natural carnosine derivative, shows an anti-hyperuricaemic effect, which was closely associated with an increasing abundance of clostridium and lactobacillus in the gut. Importantly, anserine-mediated regulation of uric acid transporters ABCG2, URAT1, and GLUT9 is dependent on intestinal flora (80). In addition, intestinal ABCG2 expression was significantly suppressed by gingko biloba leaf extract (GLE) administration, which is closely related to decreased populations of proteobacteria and deferribacteres at the phylum level. It is worth noting that GLE treatment did not affect ABCG2 expression, but treatment with the lysates of GLE-treated mouse stool significantly suppressed ABCG2 expression. These findings reveal a role for intestinal flora in regulating ABCG2 expression (81). Not only does the intestinal flora plays an important role in intestinal immune homeostasis, studies have also shown it can regulate uric acid transporters through its metabolites. In SCFAs treated rats, the expression and function of intestinal ABCG2 were increased. Similar results were also observed in mouse primary enterocytes and Caco-2 cells treated with SCFAs (82). These findings showed that microbiota has a regulatory effect on expression of uric acid transporters.

In keeping with *in vitro* and animal studies, data from large-scale metagenome genome-wide association studies (mgGWAS) for the oral microbiome revealed unequivocal human genetic loci associated with the oral microbiome, including uric acid transporter SLC2A9. SLC2A9 showed a strong correlation with species-level clusters belonging to oribacterium and lanchnoanaerobaculum in tongue dorsum samples (83). A previous study reported that oral cavity and stool bacteria overlapped in more than 45% of subjects (84). Interestingly, the tongue dorsum microbiota related gene SLC2A9 was correlated with the abundance of Bifidobacterium animalis in the gut (83). These results demonstrated that gut microbial diversity also has an impact on uric acid transporters.

## Summary

Currently uric acid lowering drugs are mainly achieved through the kidneys, while the intestine is the second largest uric acid excretion organ. The decrease in intestinal uric acid excretion will increase the burden on the kidneys. At present, the intestinal excretion of uric acid has been used as a new direction for the treatment of gout and hyperuricemia, which can avoid side effects such as aggravating kidney damage and urinary tract stone formation. In summary, there is a bright prospect for drug and gut microbiota research targeting intestinal uric acid transporters to treat hyperuricemia and gout (**Figure 3**). However, there are scarce studies about the association between intestinal flora and uric acid transporters, and the specific mechanism of the interaction between these need to be further elucidated.

## Author Contributions

HY, NL, and JC reviewed the literature and wrote the first draft. HY and JC reviewed the literature and finalized the manuscript. All authors have read and approved the final manuscript.

## Conflict of Interest

The authors declare that the research was conducted in the absence of any commercial or financial relationships that could be construed as a potential conflict of interest.

## Publisher’s Note

All claims expressed in this article are solely those of the authors and do not necessarily represent those of their affiliated organizations, or those of the publisher, the editors and the reviewers. Any product that may be evaluated in this article, or claim that may be made by its manufacturer, is not guaranteed or endorsed by the publisher.

## References

[1] Richette P Bardin T. Gout. Lancet (2010) 375:318–28. doi: 10.1016/S0140-6736(09)60883-7 19692116

[2] Maiuolo J Oppedisano F Gratteri S Muscoli C Mollace V. Regulation of Uric Acid Metabolism and Excretion. Int J Cardiol (2016) 213:8–14. doi: 10.1016/j.ijcard.2015.08.109 26316329

[3] Dalbeth N Merriman TR Stamp LK. Gout. Lancet (2016) 388:2039–52. doi: 10.1016/S0140-6736(16)00346-9 27112094

[4] Lv S Liu W Zhou Y Liu Y Shi D Zhao Y. Hyperuricemia and Severity of Coronary Artery Disease: An Observational Study in Adults 35 Years of Age and Younger With Acute Coronary Syndrome. Cardiol J (2019) 26:275–82. doi: 10.5603/CJ.a2018.0022 PMC8086668 29512091

[5] Chang CC Wu CH Liu LK Chou RH Kuo CS Huang PH. Association Between Serum Uric Acid and Cardiovascular Risk in Nonhypertensive and Nondiabetic Individuals: The Taiwan I-Lan Longitudinal Aging Study. Sci Rep (2018) 8:5234. doi: 10.1038/s41598-018-22997-0 29588485 PMC5869680

[6] Stamp LK Chapman PT. Gout and Its Comorbidities: Implications for Therapy. Rheumatology (2013) 52:34–44. doi: 10.1093/rheumatology/kes211 22949727

[7] Sorensen LB. Role of the Intestinal Tract in the Elimination of Uric Acid. Arthritis Rheum (1965) 8:694–706. doi: 10.1002/art.1780080429 5859543

[8] Boss GR Seegmiller JE. Hyperuricemia and Gout. Classification, Complications and Management. N Engl J Med (1979) 300:1459–68. doi: 10.1056/NEJM197906283002604 221806

[9] Yamauchi T Ueda T. Primary Hyperuricemia Due to Decreased Renal Uric Acid Excretion. Nihon Rinsho (2008) 66:679–81. 18409514

[10] Ichida K Matsuo H Takada T Nakayama A Murakami K Shimizu T. Decreased Extra-Renal Urate Excretion Is a Common Cause of Hyperuricemia. Nat Commun (2012) 3:764. doi: 10.1038/ncomms1756 22473008 PMC3337984

[11] Strilchuk L Fogacci F Cicero AF. Safety and Tolerability of Available Urate-Lowering Drugs: A Critical Review. Expert Opin Drug Saf (2019) 18:261–71. doi: 10.1080/14740338.2019.1594771 30915866

[12] Cao ZH Wei ZY Zhu QY Zhang JY Yang L Qin SY. HLA-B*58:01 Allele Is Associated With Augmented Risk for Both Mild and Severe Cutaneous Adverse Reactions Induced by Allopurinol in Han Chinese. Pharmacogenomics (2012) 13:1193–201. doi: 10.2217/pgs.12.89 22909208

[13] White WB Saag KG Becker MA Borer JS Gorelick PB Whelton A. Cardiovascular Safety of Febuxostat or Allopurinol in Patients With Gout. N Engl J Med (2018) 378:1200–10. doi: 10.1056/NEJMoa1710895 29527974

[14] Becker MA Baraf HS Yood RA Dillon A Vazquez-Mellado J Ottery FD. Long-Term Safety of Pegloticase in Chronic Gout Refractory to Conventional Treatment. Ann Rheum Dis (2013) 72:1469–74. doi: 10.1136/annrheumdis-2012-201795 PMC3756467 23144450

[15] Pui CH. Rasburicase: A Potent Uricolytic Agent. Expert Opin Pharmacother (2002) 3:433–42. doi: 10.1517/14656566.3.4.433 11934348

[16] Tausche AK Jansen TL Schroder HE Bornstein SR Aringer M Muller-Ladner U. Gout–current Diagnosis and Treatment. Dtsch Arztebl Int (2009) 106:549–55. doi: 10.3238/arztebl.2009.0549 PMC2754667 19795010

[17] Boger WP Strickland SC. Probenecid (Benemid); Its Uses and Side-Effects in 2,502 Patients. AMA Arch Intern Med (1955) 95:83–92. doi: 10.1001/archinte.1955.00250070099012 13217507

[18] Tausche A-K Alten R Dalbeth N Kopicko J Fung M Adler S. Lesinurad Monotherapy in Gout Patients Intolerant to a Xanthine Oxidase Inhibitor: A 6 Month Phase 3 Clinical Trial and Extension Study. Rheumatology (2017) 56:2170–8. doi: 10.1093/rheumatology/kex350 29029210

[19] Alvarez-Lario B Macarron-Vicente J. Uric Acid and Evolution. Rheumatol (Oxford) (2010) 49:2010–5. doi: 10.1093/rheumatology/keq204 20627967

[20] Keenan RT. The Biology of Urate. Semin Arthritis Rheum (2020) 50:S2–S10. doi: 10.1016/j.semarthrit.2020.04.007 32620198

[21] Wu XW Lee CC Muzny DM Caskey CT. Urate Oxidase: Primary Structure and Evolutionary Implications. Proc Natl Acad Sci USA (1989) 86:9412–6. doi: 10.1073/pnas.86.23.9412 PMC298506 2594778

[22] Lipkowitz MS. Regulation of Uric Acid Excretion by the Kidney. Curr Rheumatol Rep (2012) 14:179–88. doi: 10.1007/s11926-012-0240-z 22359229

[23] Fathallah-Shaykh SA Cramer MT. Uric Acid and the Kidney. Pediatr Nephrol (2014) 29:999–1008. doi: 10.1007/s00467-013-2549-x 23824181

[24] Li C Li Z Liu S Wang C Han L Cui L. Genome-Wide Association Analysis Identifies Three New Risk Loci for Gout Arthritis in Han Chinese. Nat Commun (2015) 6:7041. doi: 10.1038/ncomms8041 25967671 PMC4479022

[25] Kottgen A Albrecht E Teumer A Vitart V Krumsiek J Hundertmark C. Genome-Wide Association Analyses Identify 18 New Loci Associated With Serum Urate Concentrations. Nat Genet (2013) 45:145–54. doi: 10.1038/ng.2500 PMC3663712 23263486

[26] Major TJ Dalbeth N Stahl EA Merriman TR. An Update on the Genetics of Hyperuricaemia and Gout. Nat Rev Rheumatol (2018) 14:341–53. doi: 10.1038/s41584-018-0004-x 29740155

[27] Xu X Li C Zhou P Jiang T. Uric Acid Transporters Hiding in the Intestine. Pharm Biol (2016) 54:3151–5. doi: 10.1080/13880209.2016.1195847 27563755

[28] Matsuo H Takada T Ichida K Nakamura T Nakayama A Ikebuchi Y. Common Defects of ABCG2, a High-Capacity Urate Exporter, Cause Gout: A Function-Based Genetic Analysis in a Japanese Population. Sci Transl Med (2009) 1:5ra11. doi: 10.1126/scitranslmed.3000237 20368174

[29] Huls M Brown CDA Windass AS Sayer R van den Heuvel JJMW Heemskerk S. The Breast Cancer Resistance Protein Transporter ABCG2 Is Expressed in the Human Kidney Proximal Tubule Apical Membrane. Kidney Int (2008) 73:220–5. doi: 10.1038/sj.ki.5002645 17978814

[30] Maliepaard M Scheffer GL Faneyte IF van Gastelen MA Pijnenborg A Schinkel AH. Subcellular Localization and Distribution of the Breast Cancer Resistance Protein Transporter in Normal Human Tissues. Cancer Res (2001) 61:3458–64. 11309308

[31] Dehghan A Köttgen A Yang Q Hwang S-J Kao WHL Rivadeneira F. Association of Three Genetic Loci With Uric Acid Concentration and Risk of Gout: A Genome-Wide Association Study. Lancet (2008) 372:1953–61. doi: 10.1016/S0140-6736(08)61343-4 PMC2803340 18834626

[32] Woodward OM Kottgen A Coresh J Boerwinkle E Guggino WB Kottgen M. Identification of a Urate Transporter, ABCG2, With a Common Functional Polymorphism Causing Gout. Proc Natl Acad Sci USA (2009) 106:10338–42. doi: 10.1073/pnas.0901249106 PMC2700910 19506252

[33] Hosomi A Nakanishi T Fujita T Tamai I. Extra-Renal Elimination of Uric Acid *via* Intestinal Efflux Transporter BCRP/Abcg2. PloS One (2012) 7:e30456. doi: 10.1371/journal.pone.0030456 22348008 PMC3277506

[34] Chen M Lu X Lu C Shen N Jiang Y Chen M. Soluble Uric Acid Increases PDZK1 and ABCG2 Expression in Human Intestinal Cell Lines *via* the TLR4-NLRP3 Inflammasome and PI3K/Akt Signaling Pathway. Arthritis Res Ther (2018) 20:20. doi: 10.1186/s13075-018-1512-4 29415757 PMC5803867

[35] Shimizu T Sugiura T Wakayama T Kijima A Nakamichi N Iseki S. PDZK1 Regulates Breast Cancer Resistance Protein in Small Intestine. Drug Metab Dispos (2011) 39:2148–54. doi: 10.1124/dmd.111.040295 21816982

[36] Kondo C Suzuki H Itoda M Ozawa S Sawada J Kobayashi D. Functional Analysis of SNPs Variants of BCRP/Abcg2. Pharm Res (2004) 21:1895–903. doi: 10.1023/B:PHAM.0000045245.21637.d4 15553238

[37] Nakamura M Fujita K Toyoda Y Takada T Hasegawa H Ichida K. Investigation of the Transport of Xanthine Dehydrogenase Inhibitors by the Urate Transporter ABCG2. Drug Metab Pharmacokinet (2018) 33:77–81. doi: 10.1016/j.dmpk.2017.11.002 29342419

[38] Woodward OM Tukaye DN Cui J Greenwell P Constantoulakis LM Parker BS. Gout-Causing Q141K Mutation in ABCG2 Leads to Instability of the Nucleotide-Binding Domain and can be Corrected With Small Molecules. Proc Natl Acad Sci USA (2013) 110:5223–8. doi: 10.1073/pnas.1214530110 PMC3612674 23493553

[39] Vitart V Rudan I Hayward C Gray NK Floyd J Palmer CN. SLC2A9 Is a Newly Identified Urate Transporter Influencing Serum Urate Concentration, Urate Excretion and Gout. Nat Genet (2008) 40:437–42. doi: 10.1038/ng.106 18327257

[40] Merriman TR. An Update on the Genetic Architecture of Hyperuricemia and Gout. Arthritis Res Ther (2015) 17:98. doi: 10.1186/s13075-015-0609-2 25889045 PMC4392805

[41] Caulfield MJ Munroe PB O’Neill D Witkowska K Charchar FJ Doblado M. SLC2A9 Is a High-Capacity Urate Transporter in Humans. PloS Med (2008) 5:e197. doi: 10.1371/journal.pmed.0050197 18842065 PMC2561076

[42] Zhang X Yang X Wang M Li X Xia Q Xu S. Association Between SLC2A9 (GLUT9) Gene Polymorphisms and Gout Susceptibility: An Updated Meta-Analysis. Rheumatol Int (2016) 36:1157–65. doi: 10.1007/s00296-016-3503-6 27255295

[43] DeBosch BJ Kluth O Fujiwara H Schurmann A Moley K. Early-Onset Metabolic Syndrome in Mice Lacking the Intestinal Uric Acid Transporter SLC2A9. Nat Commun (2014) 5:4642. doi: 10.1038/ncomms5642 25100214 PMC4348061

[44] Prestin K Wolf S Feldtmann R Hussner J Geissler I Rimmbach C. Transcriptional Regulation of Urate Transportosome Member SLC2A9 by Nuclear Receptor HNF4alpha. Am J Physiol Renal Physiol (2014) 307:F1041–51. doi: 10.1152/ajprenal.00640.2013 25209865

[45] Berger J Moller DE. The Mechanisms of Action of PPARs. Annu Rev Med (2002) 53:409–35. doi: 10.1146/annurev.med.53.082901.104018 11818483

[46] Phipps-Green AJ Merriman ME Topless R Altaf S Montgomery GW Franklin C. Twenty-Eight Loci That Influence Serum Urate Levels: Analysis of Association With Gout. Ann Rheum Dis (2016) 75:124–30. doi: 10.1136/annrheumdis-2014-205877 25187157

[47] Nakayama A Matsuo H Shimizu T Ogata H Takada Y Nakashima H. A Common Missense Variant of Monocarboxylate Transporter 9 (MCT9/SLC16A9) Gene Is Associated With Renal Overload Gout, But Not With All Gout Susceptibility. Hum Cell (2013) 26:133–6. doi: 10.1007/s13577-013-0073-8 PMC3844819 23990105

[48] Togawa N Miyaji T Izawa S Omote H Moriyama Y. A Na+-Phosphate Cotransporter Homologue (SLC17A4 Protein) Is an Intestinal Organic Anion Exporter. Am J Physiol Cell Physiol (2012) 302:C1652–60. doi: 10.1152/ajpcell.00015.2012 22460716

[49] H.M.P.R.N.C. Integrative. The Integrative Human Microbiome Project. Nature (2019) 569:641–8. doi: 10.1038/s41586-019-1238-8 PMC6784865 31142853

[50] Hall JA Bouladoux N Sun CM Wohlfert EA Blank RB Zhu Q. Commensal DNA Limits Regulatory T Cell Conversion and Is a Natural Adjuvant of Intestinal Immune Responses. Immunity (2008) 29:637–49. doi: 10.1016/j.immuni.2008.08.009 PMC2712925 18835196

[51] Pickard JM Zeng MY Caruso R Nunez G. Gut Microbiota: Role in Pathogen Colonization, Immune Responses, and Inflammatory Disease. Immunol Rev (2017) 279:70–89. doi: 10.1111/imr.12567 28856738 PMC5657496

[52] Xing SC Meng DM Chen Y Jiang G Liu XS Li N. Study on the Diversity of Bacteroides and Clostridium in Patients With Primary Gout. Cell Biochem Biophys (2015) 71:707–15. doi: 10.1007/s12013-014-0253-5 25344643

[53] Shao T Shao L Li H Xie Z He Z Wen C. Combined Signature of the Fecal Microbiome and Metabolome in Patients With Gout. Front Microbiol (2017) 8:268. doi: 10.3389/fmicb.2017.00268 28270806 PMC5318445

[54] Szulinska M Loniewski I van Hemert S Sobieska M Bogdanski P. Dose-Dependent Effects of Multispecies Probiotic Supplementation on the Lipopolysaccharide (LPS) Level and Cardiometabolic Profile in Obese Postmenopausal Women: A 12-Week Randomized Clinical Trial. Nutrients (2018) 10:773–85. doi: 10.3390/nu10060773 PMC6024794 29914095

[55] Wang J Chen Y Zhong H Chen F Regenstein J Hu X. The Gut Microbiota as a Target to Control Hyperuricemia Pathogenesis: Potential Mechanisms and Therapeutic Strategies. Crit Rev Food Sci Nutr (2021) 22:1–11. doi: 10.1080/10408398.2021.1874287 33480266

[56] García-Arroyo FE Gonzaga G Muñoz-Jiménez I Blas-Marron MG Silverio O Tapia E. Probiotic Supplements Prevented Oxonic Acid-Induced Hyperuricemia and Renal Damage. PloS One (2018) 13:e0202901. doi: 10.1371/journal.pone.0202901 30142173 PMC6108486

[57] Lin X Shao T Huang L Wen X Wang M Wen C. Simiao Decoction Alleviates Gouty Arthritis by Modulating Proinflammatory Cytokines and the Gut Ecosystem. Front Pharmacol (2020) 11:955. doi: 10.3389/fphar.2020.00955 32670069 PMC7327538

[58] Buzard J Bishop C Talbott JH. Recovery in Humans of Intravenously Injected Isotopic Uric Acid. J Biol Chem (1952) 196:179–84. doi: 10.1016/S0021-9258(18)55717-3 12980954

[59] Crane JK. Role of Host Xanthine Oxidase in Infection Due to Enteropathogenic and Shiga-Toxigenic Escherichia Coli. Gut Microbes (2013) 4:388–91. doi: 10.4161/gmic.25584 PMC3839983 23811846

[60] Yun Y Yin H Gao Z Li Y Gao T Duan J. Intestinal Tract Is an Important Organ for Lowering Serum Uric Acid in Rats. PloS One (2017) 12:e0190194. doi: 10.1371/journal.pone.0190194 29267361 PMC5739491

[61] Mendez-Salazar EO Martinez-Nava GA. Uric Acid Extrarenal Excretion: The Gut Microbiome as an Evident Yet Understated Factor in Gout Development. Rheumatol Int (2021). doi: 10.1007/s00296-021-05007-x 34586473

[62] Ramazzina I Costa R Cendron L Berni R Peracchi A Zanotti G. An Aminotransferase Branch Point Connects Purine Catabolism to Amino Acid Recycling. Nat Chem Biol (2010) 6:801–6. doi: 10.1038/nchembio.445 20852637

[63] Li M Yang D Mei L Yuan L Xie A Yuan J. Screening and Characterization of Purine Nucleoside Degrading Lactic Acid Bacteria Isolated From Chinese Sauerkraut and Evaluation of the Serum Uric Acid Lowering Effect in Hyperuricemic Rats. PloS One (2014) 9:e105577. doi: 10.1371/journal.pone.0105577 25184445 PMC4153548

[64] Samuel BS Shaito A Motoike T Rey FE Backhed F Manchester JK. Effects of the Gut Microbiota on Host Adiposity Are Modulated by the Short-Chain Fatty-Acid Binding G Protein-Coupled Receptor, Gpr41. Proc Natl Acad Sci USA (2008) 105:16767–72. doi: 10.1073/pnas.0808567105 PMC2569967 18931303

[65] Hsu YJ Chiu CC Li YP Huang WC Huang YT Huang CC. Effect of Intestinal Microbiota on Exercise Performance in Mice. J Strength Cond Res (2015) 29:552–8. doi: 10.1519/JSC.0000000000000644 25144131

[66] Guo Z Zhang J Wang Z Ang KY Huang S Hou Q. Intestinal Microbiota Distinguish Gout Patients From Healthy Humans. Sci Rep (2016) 6:20602. doi: 10.1038/srep20602 26852926 PMC4757479

[67] Chu Y Sun S Huang Y Gao Q Xie X Wang P. Metagenomic Analysis Revealed the Potential Role of Gut Microbiome in Gout. NPJ Biofilms Microbiomes (2021) 7:66. doi: 10.1038/s41522-021-00235-2 34373464 PMC8352958

[68] Nieuwdorp M Gilijamse PW Pai N Kaplan LM. Role of the Microbiome in Energy Regulation and Metabolism. Gastroenterology (2014) 146:1525–33. doi: 10.1053/j.gastro.2014.02.008 24560870

[69] Tremaroli V Backhed F. Functional Interactions Between the Gut Microbiota and Host Metabolism. Nature (2012) 489:242–9. doi: 10.1038/nature11552 22972297

[70] Macia L Tan J Vieira AT Leach K Stanley D Luong S. Metabolite-Sensing Receptors GPR43 and GPR109A Facilitate Dietary Fibre-Induced Gut Homeostasis Through Regulation of the Inflammasome. Nat Commun (2015) 6:6734. doi: 10.1038/ncomms7734 25828455

[71] Kim M Qie Y Park J Kim CH. Gut Microbial Metabolites Fuel Host Antibody Responses. Cell Host Microbe (2016) 20:202–14. doi: 10.1016/j.chom.2016.07.001 PMC4982788 27476413

[72] Vieira AT Galvao I Macia LM Sernaglia EM Vinolo MA Garcia CC. Dietary Fiber and the Short-Chain Fatty Acid Acetate Promote Resolution of Neutrophilic Inflammation in a Model of Gout in Mice. J Leukoc Biol (2017) 101:275–84. doi: 10.1189/jlb.3A1015-453RRR 27496979

[73] Lyu LC Hsu CY Yeh CY Lee MS Huang SH Chen CL. A Case-Control Study of the Association of Diet and Obesity With Gout in Taiwan. Am J Clin Nutr (2003) 78:690–701. doi: 10.1093/ajcn/78.4.690 14522726

[74] Tan J McKenzie C Potamitis M Thorburn AN Mackay CR Macia L. The Role of Short-Chain Fatty Acids in Health and Disease. Adv Immunol (2014) 121:91–119. doi: 10.1016/B978-0-12-800100-4.00003-9 24388214

[75] Sun M Wu W Liu Z Cong Y. Microbiota Metabolite Short Chain Fatty Acids, GPCR, and Inflammatory Bowel Diseases. J Gastroenterol (2017) 52:1–8. doi: 10.1007/s00535-016-1242-9 27448578 PMC5215992

[76] Furusawa Y Obata Y Fukuda S Endo TA Nakato G Takahashi D. Commensal Microbe-Derived Butyrate Induces the Differentiation of Colonic Regulatory T Cells. Nature (2013) 504:446–50. doi: 10.1038/nature12721 24226770

[77] Singh N Gurav A Sivaprakasam S Brady E Padia R Shi H. Activation of Gpr109a, Receptor for Niacin and the Commensal Metabolite Butyrate, Suppresses Colonic Inflammation and Carcinogenesis. Immunity (2014) 40:128–39. doi: 10.1016/j.immuni.2013.12.007 PMC4305274 24412617

[78] Vinolo MA Rodrigues HG Hatanaka E Sato FT Sampaio SC Curi R. Suppressive Effect of Short-Chain Fatty Acids on Production of Proinflammatory Mediators by Neutrophils. J Nutr Biochem (2011) 22:849–55. doi: 10.1016/j.jnutbio.2010.07.009 21167700

[79] Puertollano E Kolida S Yaqoob P. Biological Significance of Short-Chain Fatty Acid Metabolism by the Intestinal Microbiome. Curr Opin Clin Nutr Metab Care (2014) 17:139–44. doi: 10.1097/MCO.0000000000000025 24389673

[80] Han J Wang Z Lu C Zhou J Li Y Ming T. The Gut Microbiota Mediates the Protective Effects of Anserine Supplementation on Hyperuricaemia and Associated Renal Inflammation. Food Funct (2021) 12:9030–42. doi: 10.1039/D1FO01884A 34382991

[81] Kim JK Choi MS Kim JY Yu JS Seo JI Yoo HH. Ginkgo Biloba Leaf Extract Suppresses Intestinal Human Breast Cancer Resistance Protein Expression in Mice: Correlation With Gut Microbiota. BioMed Pharmacother (2021) 140:111712. doi: 10.1016/j.biopha.2021.111712 34010745

[82] Xie QS Zhang JX Liu M Liu PH Wang ZJ Zhu L. Short-Chain Fatty Acids Exert Opposite Effects on the Expression and Function of P-Glycoprotein and Breast Cancer Resistance Protein in Rat Intestine. Acta Pharmacol Sin (2021) 42:470–81. doi: 10.1038/s41401-020-0402-x PMC8027219 32555444

[83] Liu X Tong X Zhu J Tian L Jie Z Zou Y. Metagenome-Genome-Wide Association Studies Reveal Human Genetic Impact on the Oral Microbiome. Cell Discov (2021) 7:117. doi: 10.1038/s41421-021-00356-0 34873157 PMC8648780

[84] Segata N Haake SK Mannon P Lemon KP Waldron L Gevers D. Composition of the Adult Digestive Tract Bacterial Microbiome Based on Seven Mouth Surfaces, Tonsils, Throat and Stool Samples. Genome Biol (2012) 13:R42. doi: 10.1186/gb-2012-13-6-r42 22698087 PMC3446314

